# A Double-Edged Sword: The Two Faces of PARylation

**DOI:** 10.3390/ijms23179826

**Published:** 2022-08-29

**Authors:** Mincheol Kang, Seojin Park, Seong-Hoon Park, Hee Gu Lee, Jun Hong Park

**Affiliations:** 1 Herbal Medicine Resources Research Center, Korea Institute of Oriental Medicine, Naju-si 58245, Korea; 2 Genetic and Epigenetic Toxicology Research Group, Korea Institute of Toxicology, Daejeon 34141, Korea; 3 Immunotherapy Research Center, Korea Research Institute of Bioscience and Biotechnology, Daejeon 34141, Korea

**Keywords:** PARylation, poly(ADP-ribose) polymerases (PARPs), ADP-ribose, NAD^+^, protein post-translational modifications

## Abstract

Poly ADP-ribosylation (PARylation) is a post-translational modification process. Following the discovery of PARP-1, numerous studies have demonstrated the role of PARylation in the DNA damage and repair responses for cellular stress and DNA damage. Originally, studies on PARylation were confined to PARP-1 activation in the DNA repair pathway. However, the interplay between PARylation and DNA repair suggests that PARylation is important for the efficiency and accuracy of DNA repair. PARylation has contradicting roles; however, recent evidence implicates its importance in inflammation, metabolism, and cell death. These differences might be dependent on specific cellular conditions or experimental models used, and suggest that PARylation may play two opposing roles in cellular homeostasis. Understanding the role of PARylation in cellular function is not only important for identifying novel therapeutic approaches; it is also essential for gaining insight into the mechanisms of unexplored diseases. In this review, we discuss recent reports on the role of PARylation in mediating diverse cellular functions and homeostasis, such as DNA repair, inflammation, metabolism, and cell death.

## 1. Introduction

Since the discovery of protein post-translational modifications (PTMs), PTMs have been recognized as important cellular regulatory mechanisms, and uncontrolled PTMs can induce disease or cellular abnormalities [1,2]. A PTM is a covalent process that alters the function or characteristics of a protein via adding functional groups or through the proteolytic cleavage of regulatory subunits. There are several types of PTM, which are classified based on their modification process or unique characteristics such as methylation, phosphorylation, acetylation, ubiquitylation, SUMOylation, glycosylation, and PARylation. These PTMs regulate various essential biological processes, such as gene expression, enzyme activity, protein stability, aging, and metabolism [2,3,4,5]. Phosphorylation is one of the most common PTMs, and it regulates the activity of enzymes or the function of proteins [6].

Poly ADP-ribosylation (PARylation) is another common PTM in eukaryotes [7,8]. In the last four decades, our understanding of the function of poly(ADP-ribose) polymerases (PARPs) and PARylation has greatly expanded since the discovery of PARP [7]. PARylation is classified by the number of units of ADP-ribose (PAR), as mono PARylation (MARylation), oligo PARylation, and poly PARylation [9]. PARylation is catalyzed by PARPs, comprising 17 protein families in humans and 16 members in mice [9]. PARPs have one or more additional domains required for their unique roles [10]. Among the PARPs, PARP-1, PARP-2, PARP-5a, and PARP-5b induce poly PARylation during the DNA damage response (DDR) and DNA repair [11]. Other PARPs mainly induce MARylation in the nucleus or cytoplasm, except for PARP-9 and PARP-13 [12].

PARP-1, belonging to the first PARPs family, is the most abundant. It is the founding member of PARPs for the synthesis of ADP-ribose using nicotinamide adenine dinucleotide (NAD^+^) as a substrate [13]. PARP-1 contains an N-terminal DNA-binding domain, nuclear localization signal, central automodification domain, and a C-terminal catalytic domain. PARP-1 is ubiquitously expressed in mammalian cells, and is localized in the nucleus. PARP-1 is activated by the DDR, and is responsible for the majority (~90%) of global PAR synthesis following DNA strand breakage. PARPs covalently combine with the poly(ADP-ribose) (PAR) unit on the carboxyl group of acidic residues, such as glutamate, aspartate, and/or lysine residues in the target protein. PAR polymers may alter chromatin structure or disrupt protein–protein or DNA interactions due to their negative charge [14]. A recent proteomics study indicated that many DDR and DNA repair proteins are PARylated during their respective processes [15]. Interestingly, PARP-1 and PARP-2 can auto-PARylate themselves.

Historically, studies on PARylation have mainly focused on the DDR and repair pathways. Under DNA damage conditions, PARPs move to the DNA damage site and auto-PARylate themselves. This phenomenon induces chromatin remodeling and recruits other DNA repair proteins. However, recent studies have suggested novel roles for PARylation in inflammation, metabolism, and cell death [16,17,18,19]. In this review, we discuss recent findings on the roles of PARylation. We also discuss the positive and negative roles of PARylation and the possibility of clinical applications. Finally, we identify important questions and issues pertaining to the role of PARylation in cellular function and homeostasis that remain to be addressed.

## 2. Role of PARylation in DNA Damage Response and Repair

**Figure 1:**
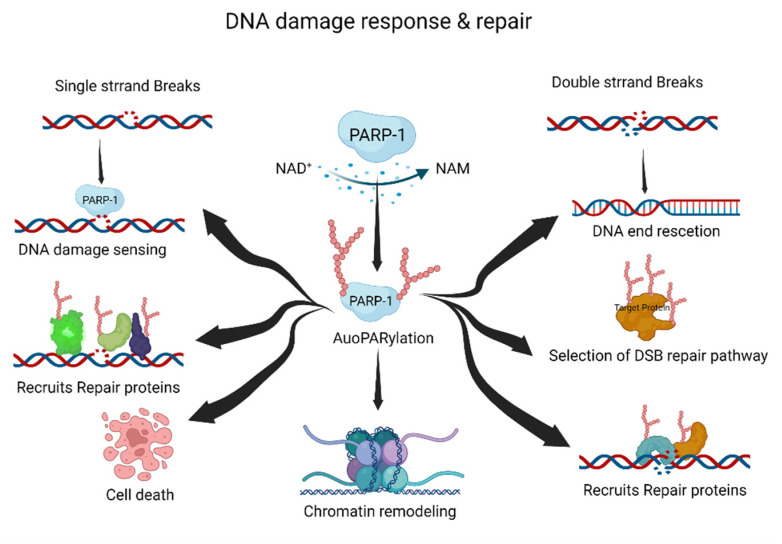
The roles of PARylation in DNA damage response and repair. PARylation acts as an initiator and/or selection effector of the DNA damage response and repair processes. NAD^+^, nicotinamide adenine dinucleotide; NAM, nicotinamide. This figure was created using BioRender.

PARP-1, a DNA-damage-sensing protein, can attach a negatively charged PAR to itself or target proteins. This process involves the consumption of large amounts of cellular NAD^+^ under DNA damage [20,21]. PARylation has multiple roles in the DDR and DNA repair pathways, including the repair of single-strand breaks (SSBs), double-strand breaks (DSBs), DNA replication forks, and chromatin structures (Figure 1) [20,22,23].

Typically, PARylation is induced by cellular stress and DNA damage [24,25]. When DNA damage occurs, the DDR recruits PARP-1 to the DNA damage site. Recruited PARP-1 induces the auto-PARylation and PARylation of other proteins, including DNA repair and chromatin structure proteins. These processes are essential for the DDR and DNA damage repair pathways. The PARylated PARP-1 at the DNA damage site is composed of linear or branched repeats of ADP-ribose. Repeating ADP-ribose units are combined through ribose–ribose glycosidic bonds to generate linear repeats of ADP-ribose, and the linear chain is branched every 20–50 ADP-ribose repeat units [8]. DNA repair proteins and PARylated proteins bind to PAR on auto-PARylated PARP-1 through noncovalent interactions [26]. The linear or branched repeats of ADP-ribose recruit more DNA repair or PARylated proteins [11,24]. Interestingly, the branching or chain length of PARylation influences its functions in cellular physiology and stress responses [27].

SSBs are the most frequently generated form of DNA damage, and are caused by direct attacks from intracellular metabolites and/or spontaneous DNA decay. Therefore, the SSB repair pathway is one of the most important repair pathways [28]. PARP-1 rapidly detects SSBs and binds to the SSB region. Auto-PARylated PARP-1 then recruits X-ray repair cross-complementing protein 1 (XRCC1) for SSB repair. XRCC1 is the main effector of the SSB repair pathway, and acts as a scaffold for SSB repair proteins such as DNA ligase 3 and DNA polymerase β [29]. The mutation of XRCC1 in human and mouse models inhibits the SSB repair pathway, resulting in neuropathological defects with hyper-PARylation [30]. Hyper-PARylation in the unrepaired SSB repair pathway leads to the depletion of cellular NAD levels, which results in cell death. Nucleotide excision repair (NER) is also regulated by PARylation. The major NER repair pathway for bulky DNA lesions is induced by various mutagenic agents, such as ultraviolet (UV) irradiation [31,32]. The protein–protein complex of xeroderma pigmentosum C (XPC)–RAD23B recognizes UV damage and initiates the NER pathway [33]. XPC–RAD23B binds to PAR and PARylation of XPC–RAD23B modulates the recognition of UV damage sites [34]. DNA-damage-binding protein 2 (DDB2) stimulates histone PARylation, which induces nucleosome displacement and triggers the NER pathway [35]. Thus, PARylation plays an essential role in NER initiation and efficiency.

DSBs can be induced by various DNA-damaging agents, such as ionizing irradiation, chemicals, DNA replication, and DNA repair processes [36]. Homologous recombination (HR) and non-homologous end joining (NHEJ) are the major DSB repair pathways. Pathway selection is determined by the cell cycle stage and chromatin condition of DSBs. PARP-1 recognizes DSBs and induces PARylation, leading to the recruitment of DSB repair proteins [21,37]. Meiotic recombination 11 (MRE11) is recruited at the DSB site by PARylation, and is involved in DNA end resection and HR selection [38]. The function of breast cancer type 1 (BRCA1) is regulated by PARylation [39,40]. BRCA1 is recruited by PARylation at the DSB site, and PARylated BRCA1 regulates DNA recombination [41]. Furthermore, PARylation inhibits hyper-resected DNA DSB. The inhibition of PARylation induces hyper-resected DNA DSB due to the PARylation-mediated recruitment of Ku and p53-binding protein 1 (p53BP1) to DNA damage sites [37]. The role of PARylation in NHEJ is not as well explored as that in HR; however, some functions have been proposed. PARylation may enhance the recruitment of NHEJ repair proteins at DSB sites and promote NHEJ [42]. Therefore, PARylation is important for the initiation and accuracy of DSB repair.

Multiple cellular processes need to be coordinated during DNA damage repair. To regulate cellular processes such as transcription and replication, recruited chromatin remodelers control chromatin relaxation or condensation through PARylation [14,23,43,44,45]. PARP-1 and PARylation provide a scaffold for the recruitment of various DNA repair proteins and chromatin remodelers. PARylation of histone core proteins, including H2A, H2B, H3, and H4, may induce nucleosome disassembly, resulting in chromatin relaxation [45]. Proteins are then recruited by PARylation at the DNA damage site [23]. For example, PARylation is amplified in liver cancer 1 (ALC1/CHD1L) at the nucleosome disassembly site, and ALC1/CHD1L enhances the accessibility of DNA repair proteins to DNA damage sites [46]. Transcription repression factors are recruited via PARylation. The nucleosome remodeling and deacetylase complex, chromodomain helicase DNA-binding protein 4, and metastasis-associated 1, are recruited by PARylation [47]. PARylation removes nascent RNA and elongates RNA polymerase at the DNA damage site. Furthermore, PARylated PARP-1 binds to linker DNA, which induces chromatin condensation [23].

Following chromatin remodeling and the recruitment of DNA repair proteins to damaged sites, PAR is quickly released from the chromatin. Rapid PAR degradation is essential for cellular homeostasis and genomic stability. Hyper-PARylation or accumulation of PAR in chromatin induces genomic instability and/or cell death [48]. PARylated PARP-1 dissociates PARP-1 from the DNA damage site [49]. Catabolic enzymes such as poly(ADP-ribose) glycohydrolase (PARG), ADP-ribosyl hydrolase 3 (ADH3), and ADP-ribosyl protein lyase degrade PAR in the chromatin or nucleus [50,51,52]. These phenomena suggest that PARylation induces chromatin remodeling to increase the efficiency and accuracy of DNA repair in the DDR and DNA damage repair pathways.

## 3. Role of PARylation in Inflammation

Inflammation, a biological reaction in living organisms, is a defense mechanism that regenerates damaged cells and tissues. It utilizes immune cells and several mediators to aid recovery from damage caused by harmful stimuli. However, if inflammation is not controlled and/or a chronic inflammatory environment develops, inflammation may be a risk factor for diseases, including sepsis and autoimmune disorders [53]. Inflammatory reactions occur in various contexts, such as in tumors, viral infections, metabolic diseases, and neurodegenerative diseases; they are often accompanied by PARylation [16]. PARylation regulates the level of inducible nitric oxide synthase (iNOS), and PARP inhibitors suppress PARylation and iNOS expression, which are involved in systemic inflammatory responses [54].

**Figure 2:**
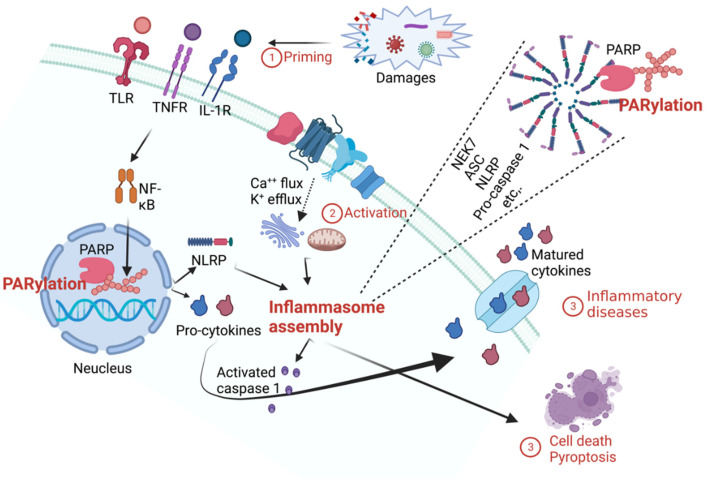
Schematic diagram of the relationships between PARylation and the inflammasome. PARylation is primed by damage signals, such as microbial molecules or endogenous cytokines, and induces the expression of NLRP and pro-cytokines through activation of the transcription factor NF-κB. PARylation of NF-κB regulates nuclear retention; in addition, PARylation may regulate the expression of inflammatory genes and the inflammatory process. During the inflammasome activation phase, mitochondrial dysfunction is induced by Ca^++^ signaling and is involved in inflammasome assembly with K^+^ efflux. It positively regulates NLRP inflammasome formation via NLRP PARylation. These processes produce pro-inflammatory cytokines that lead to cell death and inflammatory diseases. TLR, toll-like receptor; TNFR, tumor necrosis factor receptor; IL-1R, interleukin 1 receptor; NF-κB, nuclear factor kappa B; NLRP, nucleotide-binding oligomerization domain, leucine-rich repeat and pyrin domain-containing protein; NEK7, NIMA related kinase 7; ACS, apoptosis-associated speck-like protein containing a CARD. This figure was created using BioRender.

Several studies have elucidated the relationships between PARylation and inflammasomes [17,55,56]. Dysregulation of inflammasome formation occurs in several diseases, including cancer, type 2 diabetes, chronic asthma-associated airway inflammation, Huntington’s disease, and experimental autoimmune encephalomyelitis, affecting cell viability [17,55,56,57]. Inflammasome formation has been studied for two decades as an important structural feature in various inflammatory diseases. Inflammasomes are cytoplasmic oligomeric proteins responsible for the activation of the inflammatory response. When proteins involved in inflammasome formation are assembled, proinflammatory cytokines progress from an immature to a mature state. Mature interleukin 1 beta (IL-1β) and/or IL-18 cytokines regulate both innate and adaptive immunity through inflammatory signaling. Receptors such as toll-like receptors (TLRs) or tumor necrosis factor receptors (TNFRs) are activated by pathogenic factors to form inflammasomes. PARylation by PARPs positively regulates inflammasome activation [17]. The PARylation of p65-nuclear factor kappa-light-chain-enhancer of activated B cells (NF-kB) regulates its nuclear retention and interactions with exportin 1, suggesting that PARylation might modulate the expression of inflammatory genes and inflammatory processes (Figure 2) [58]. PARylation inhibitors have long been studied for the treatment of cancer and inflammatory diseases; however, more studies are required to expand our knowledge on the role(s) of PARylation in the amplification or regulation of inflammation [59]. A more in-depth study of the relationships between inflammation and PARylation will lead to the development of novel therapeutics for various diseases.

## 4. Role of PARylation in Metabolism

PARylation can directly and indirectly regulate cellular and whole-body metabolism. PARPs are widely expressed in humans and mice, including in adipose, muscle, liver, heart, pancreas, and brain. Increased PARylation in tissues or cells is representative of abnormal metabolic conditions because PARylation is an indicator of cellular stress. In addition, PARylation is an indicator of NAD^+^/NADH levels available for cellular energy sensing and signaling. Finally, PARylation has unique functions in metabolic organs and tissues. 

**Figure 3:**
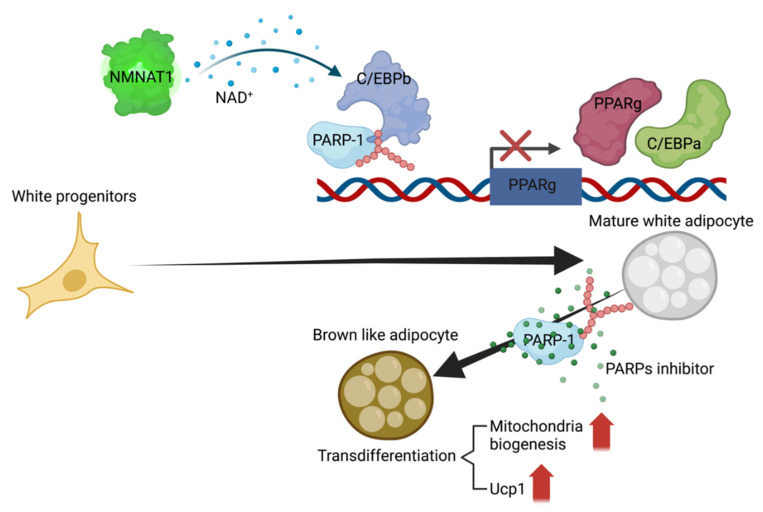
PARylation plays roles in adipogenesis. PARylation of C/EBPb blocks the expression of adipogenic regulator genes. Inhibition of PARylation induces transdifferentiation in white adipogenesis models. NMNAT-1, nicotinamide mononucleotide adenylyltransferase 1; NAD^+^, nicotinamide adenine dinucleotide; C/EBPb, CCAAT/enhancer-binding protein b; PARP-1, poly(ADP-ribose) polymerase 1; PPARg, peroxisome proliferator-activated g; C/EBPa, CCAAT/enhancer-binding protein a; Ucp1, uncoupling protein 1. This figure was created using BioRender.

Adipocytes and adipose tissues are central to metabolism. The main function of adipocytes is to control energy storage and expenditure [60]. Adipocytes are classified according to their function as white, beige, and brown adipocytes. White adipocytes directly regulate glucose and fatty acid homeostasis, and indirectly control whole-body metabolism by secreting adipokines. In contrast, brown adipocytes regulate energy expenditure through thermogenesis. Beige adipocytes can change their functions and roles according to whole-body metabolic homeostasis. However, the role of PARylation in adipogenesis remains controversial. The auto-PARylation of PARP-1 is dependent on the adipogenesis phase [18]. During clonal expansion in early adipogenesis, the PARylation signal decreases; it is increased during the terminal adipogenesis phase. Pharmacological inhibition of PARylation reinforces adipogenic differentiation. PARylated CCAAT/enhancer-binding protein β (C/EBPβ) can inactivate the expression of adipogenic regulator proteins, such as C/EBPα or peroxisome proliferator-activated γ (PPARγ). Blocking nicotinamide mononucleotide adenylyltransferase 1 (NMNAT-1), an NAD^+^ biosynthesis enzyme in the nucleus, activates adipogenesis [61]. Activation of cytoplasmic NMNAT-2 or blocking of nuclear NMNAT-1 blocks NAD^+^ synthesis in the nucleus and inhibits PARylation (Figure 3). In contrast, the pharmacological inhibition of PARylation suppresses adipogenesis. In addition, the inhibition of PARylation increases the activity of PPARγ in cardiomyocytes [62]. These contradictory phenomena might originate from differences in the experimental time-point (early or late adipogenesis) and/or experimental models (origin of the cell model). Therefore, PARylation regulates adipogenesis by functioning in diverse phases and roles.

Pharmacological inhibition of PARylation induces the transdifferentiation of white adipocytes into brown-like adipocytes, suggesting that PARylation might determine cellular lineages [63]. In addition, blocking PARylation induces mitochondrial biogenesis and increases the expression of uncoupling protein 1. Consistent with this, the preservation of cellular NAD^+^ levels activate the sirtuin 1–peroxisome proliferator-activated receptor gamma coactivator-1 axis pathway [64]. Therefore, PARylation is important for energy balance homeostasis and plays several roles in metabolic stress responses.

Although several tissues are involved in lipid metabolism, the liver is the primary tissue involved in cholesterol metabolism. Cholesterol is an essential molecule for steroid hormone and vitamin D synthesis. The liver synthesizes cholesterol and secretes it into the blood in the form of low-density lipoprotein (LDL). Liver X receptors (LXRs) are essential regulators of cholesterol transport; they are PARylated by PARP-1, which alters their function [65]. Inhibition of PARylation increases fatty acid oxidation and offers protection against fatty liver disease. Pharmacological inhibition of PARylation has protective effects against non-alcoholic and alcoholic hepatic steatosis induced by a high-fat diet [66]. In contrast, genetic depletion of PARP-1 increases hepatic lipid levels in high-fat-diet models [67]. These contradictory results might originate from differential genetic backgrounds in the mouse models [68,69].

In one study, DNA damage stress induced PARylation over 100-fold in a dose- and time-dependent manner; however, the level of PARylation was significantly different between cell types, mouse tissues, and individual human lymphocyte donors in LC-MS/MS analysis [70]. Among the mouse tissues, induced levels of PARylation were the highest in the heart, while they remained at similar levels in other tissues (kidney, liver, thymus, testis, and spleen). Thus, PARP expression and PARylation can regulate whole-body metabolism. In the brain, PARylation regulates neuronal cell differentiation, feeding, behavioral activity, and circadian rhythms [71,72]. PARP-1 binding and PARylation of CLOCK regulates food entrainment of peripheral circadian clocks [73]. Consistent with these results, PARylation is important for endocrine homeostasis. PARylation of PARP-1/2 regulates endocrine hormone transcription [74]. Insulin, sex hormones, and other endocrine hormones modulate PARylation [75,76]. In addition, PARylation of PARP-1/2 is a pathological sign of insulin resistance; however, this phenomenon might be related to cellular metabolic stress [77]. Metabolic phenotypes in genetic PARP-1/2 mutant mouse models are well explored; however, the roles of PARylation of PARPs and other target proteins in metabolic control require further study [18,68,69,71,73].

## 5. Role of PARylation in Cell Death

The role of PARylation in DNA repair is well-known. DNA damage by stressors induces PARylation, and synthesized PAR recruits the DNA repair protein complex to the DNA damage region [24]. Extensive DNA damage can lead to cell death due to a series of processes caused by hyper-PARylation; it was recognized as a suicide response in the early PARylation studies. Hyper-PARylation depletes NAD^+^ and increases NAD^+^ resynthesis because NAD^+^ is an essential factor for PARylation. It inhibits adenosine triphosphate (ATP) synthesis, resulting in ATP depletion. ATP and NAD^+^ are necessary for each other’s synthesis, and their depletion induces cell death [78].

**Figure 4:**
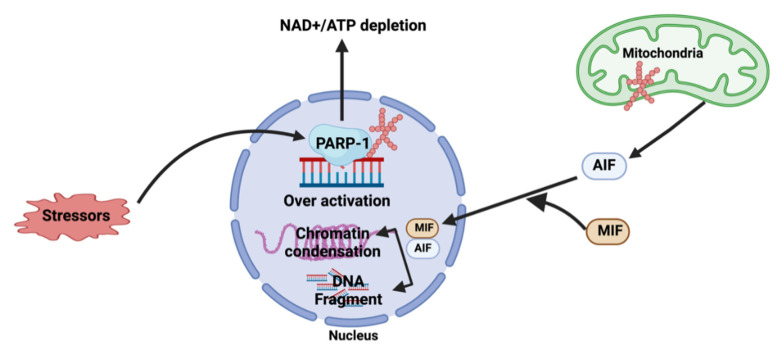
The roles of PARylation in parthanatos. PARylation is vital in the main signaling pathway of parthanatos. AIF, apoptosis-inducing factor; MIF, macrophage migration inhibitor factor. This figure was created using BioRender.

PAR polymers hypersynthesized by DNA damage responses can trigger other pathways of cell death, such as parthanatos. Parthanatos is a unique non-apoptotic programmed cell death pathway, functioning along with other novel cell death pathways [79,80]. PAR polymers from the nucleus are released into the cytoplasm. The released PAR polymers induce translocation of apoptosis-inducing factor (AIF) from the mitochondria to the nucleus, along with macrophage migration inhibitor factor (MIF). The AIF–MIF complex induces large-scale DNA fragmentation and chromatin condensation, which causes cell death (Figure 4) [19,81]. This pathway was first demonstrated by Down et al. in 2002 [82]. In the PARP-1 knockout (KO) group, N-methyl-N’-nitro-N-nitrosoguanidine (MNNG) promoted cell survival, and cells did not show AIF translocation from the mitochondria [83]. This confirmed that the PAR polymer is the main inducer of cell death via an AIF translocation-dependent mechanism. PAR polymer delivery in mouse models with genetically depleted PARP-1 led to AIF translocation and cell death. PARG, which degrades PAR polymer, prevents AIF translocation and chromatin condensation [24,82]. In PARG-null embryonic trophoblast stem (TS) cells, PAR accumulated following UV irradiation. A higher level of NAD+ was also maintained when compared to the wild type. Consistent with these results, AIF translocation to the nucleus was observed only in PARG-null TS cells [84].

The function of MIF as a nuclease in parthanatos has been identified [19]. MIF was initially discovered as a lymphokine secreted by T cells which inhibits macrophage migration, and initial research focused on its function in the immune response. It facilitates phagocytosis, stimulates cytokine expression (including tumor necrosis factor (TNF)-α, interleukin (IL)-1, IL-6, and IL-8), and antagonizes the glucocorticoid-mediated inhibition of cytokine production. It is a key mediator of the immune system and in autoimmune diseases. In addition, MIF can promote tumor growth and angiogenesis. MIF is a multifunctional molecule [19,85,86,87] which mediates PAR-related cell death. MIF cleaves genomic DNA into 20–50 kb fragments in the nucleus. The absence of MIF, inhibition of AIF binding to MIF, or mutations in the nuclease domain of MIF can block genomic DNA cleavage and prevent cell death [81]. Parthanatos is involved in the pathogenesis of various diseases, including neurodegenerative diseases such as Parkinson’s disease, Alzheimer’s disease, Huntington’s disease, amyotrophic lateral sclerosis [88], ischemic stroke [88], chronic heart failure [89], and diabetes [90,91]. Neuron cell death is one of the main causes of neurodegenerative diseases, and is induced by toxic protein aggregation. Aggregation of proteins like amyloid-beta (Aβ), tau, and α-synuclein in neuronal cells can lead to increased NOS and reactive oxygen species (ROS) production, which induces DNA damage. Hyper-PARylation indicates the progression of massive DNA damage [92], and is usually caused by overactive PARP-1. PARP-1 KO mice or PARP-1-inhibitor-treated mice demonstrated less-severe pathology when phosphorylated α-synuclein (which promotes fibril formation) was injected into the brain [93]. The parthanatos cascade may be a target for therapeutic approaches. Disturbance of the parthanatos cascade or inhibition of PARylation should be a focus of studies exploring potential therapeutic methods.

## 6. Conclusions

PARylation is a type of PTM induced by PARP activation [7,8,9]. Over the past four decades, PARylation has been extensively studied for its key roles in the DDR and DNA damage repair pathways. However, functional studies of PARylation have focused on the DDR and mechanisms of repair. There is a need for additional functional research on the recently identified biological roles of PARylation in inflammation, metabolism, and cell death.

This new knowledge of PARylation’s roles in DNA damage, inflammation, metabolism, and cell death should encourage researchers to study unexplored implications of PARylation. Almost all patients with cancer develop cancer-associated cachexia (CAC) in the last stages of tumor progression. CAC is a complicated disease that involves DNA damage, inflammation, metabolism, and cell death. For example, the PARP inhibitor Olaparib may help with cancer therapy and adipose tissue maintenance. Although PARP inhibitors are approved as therapeutics for specific cancers with defective BRCA1/2 genes, blocking PARylation can also activate adipogenesis and/or cell survival. Interestingly, PARPs and PARylation may be important in the progression of CAC, and may be targeted in novel therapeutic approaches for CAC [94,95,96,97].

Recent studies suggested that PARP-1 and PARylation may be important for aging and longevity by regulating the cellular levels of NAD^+^ [98,99,100,101,102], and because DNA damage, inflammation, metabolism, and cell death are important factors in aging and longevity. Furthermore, PARylation requires cellular NAD^+^ as substrate, and PARP-1 competes with SIRTs for cellular NAD^+^ [64]. Inhibition of PARylation could provide more activation of SIRT family proteins. Consistent with this result, many studies have demonstrated that activation of the NAD^+^–SIRT1 axis in the body or organs increases longevity and quality of life [71,103]. However, there are conflicting results with PARP-1 activation and PARylation with regards to aging and longevity. In one study, increased PARylation enhanced DNA damage repair, reducing the accumulation of DNA damage [104]. Muiras et al. also demonstrated that PARylation was correlated with longevity [105]. However, existing studies have not determined whether PARylation is a cause or consequence of aging, and a better understating of PARylation may help in identifying novel mechanisms of aging, and in finding targets to enhance longevity or quality of life.

PARylation modulates oncogenicity by interacting with various oncogenic proteins or regulating several transcription factors. Several studies have demonstrated that PARP inhibitors (PARPis) increase the sensitivity of several types of cancer cells to apoptosis [106]. It is necessary to re-evaluate the use of chemotherapeutic agents in combination with PARPis. For example, in hepatocellular carcinoma, TLR9 agonist treatment decreased PARylation of STAT3 and expression of PARP-1 [107]. Therefore, combination therapy with TLR9 agonists and PARPis may lead to improved antitumor efficacy because there are reports on the importance of KLF4 PARylation in genomic stability, carcinogenesis, and therapy. PARylation of KLF4 may be involved in oncogenic signaling, and PARPis could be alternatively used for the treatment of triple-negative breast cancers [108]. However, there are also reports that PARP-1 is associated with melanomagenesis [109]. PARP-1 promotes melanoma risk by activating oncogene transcription and cell proliferation, independently of PARylation. These studies suggest that PARP and/or PARylation have specific roles in carcinogenesis and tumor growth. Therefore, further research should focus on the roles of PARylation in carcinogenesis or the pathological progression of cancer.

Although several contrasting roles are attributed to PARylation, studies have conclusively demonstrated the importance of PARylation in DNA damage repair, inflammation, metabolism, and cell death. Essential tissues respond differently to DNA damage agents, and this may be due to differences in PARP expression and PARylation activity [110]. In DDR and DNA damage repair, PARylation is important for the efficiency and accuracy of DNA repair pathways, and can determine cell fate via hyper-PARylation. However, recent studies have demonstrated two sides of PARylation. PARylation could be a defense mechanism against chemotherapy in non-small-cell lung cancer cell lines [111]. PARP-1 suppresses the induction of colorectal cancer, but promotes the progression of colorectal cancer [112]. Increased expression of PARP-1 and PARylation may stimulate tumor growth. In addition, PARylation inhibition had a negative effect. Blocking PARylation of KLF4 suppresses the DDR and activation of telomerase reverse transcriptase [108,113]. Therefore, PARylation may be a tumorigenic factor, and these interactions require further clinical studies.

In conclusion, continuously expanding our knowledge of the role of PARylation in modulating DNA damage, inflammation, metabolism, and cell death will provide novel insights into the underlying biological and physiological mechanisms, which will aid the development of novel therapeutic approaches.
